# Correction to 'Endothelial Integrin‐Linked Kinase (ILK) Deficiency Promotes Endothelial Activation and Cardiovascular Dysfunction via Receptor Interacting Protein Kinase‐1 (RIPK1) Enriched‐Extracellular Vesicle Signalling'

**DOI:** 10.1002/jev2.70344

**Published:** 2026-08-09

Cook‐Calvete, A., M. Delgado‐Marín, S. Jorquera Ortega, et al. 2026. “Endothelial Integrin‐Linked Kinase (ILK) Deficiency Promotes Endothelial Activation and Cardiovascular Dysfunction via Receptor Interacting Protein Kinase‐1 (RIPK1) Enriched‐Extracellular Vesicle Signalling.” *Journal of Extracellular Vesicles* 15, no. 6: e70335. https://doi.org/10.1002/jev2.70335

In the originally published article, author Blanca Fernandez‐Rodriguez's name was given incorrectly as Blanca Rodríguez‐Fernandez. Additionally, Figure 6 was inadvertently published twice as Figure 6 and Figure 7. The correct Figure 7 is shown below. The online version of the article has been updated.

We apologize for this error.

**FIGURE 7:**
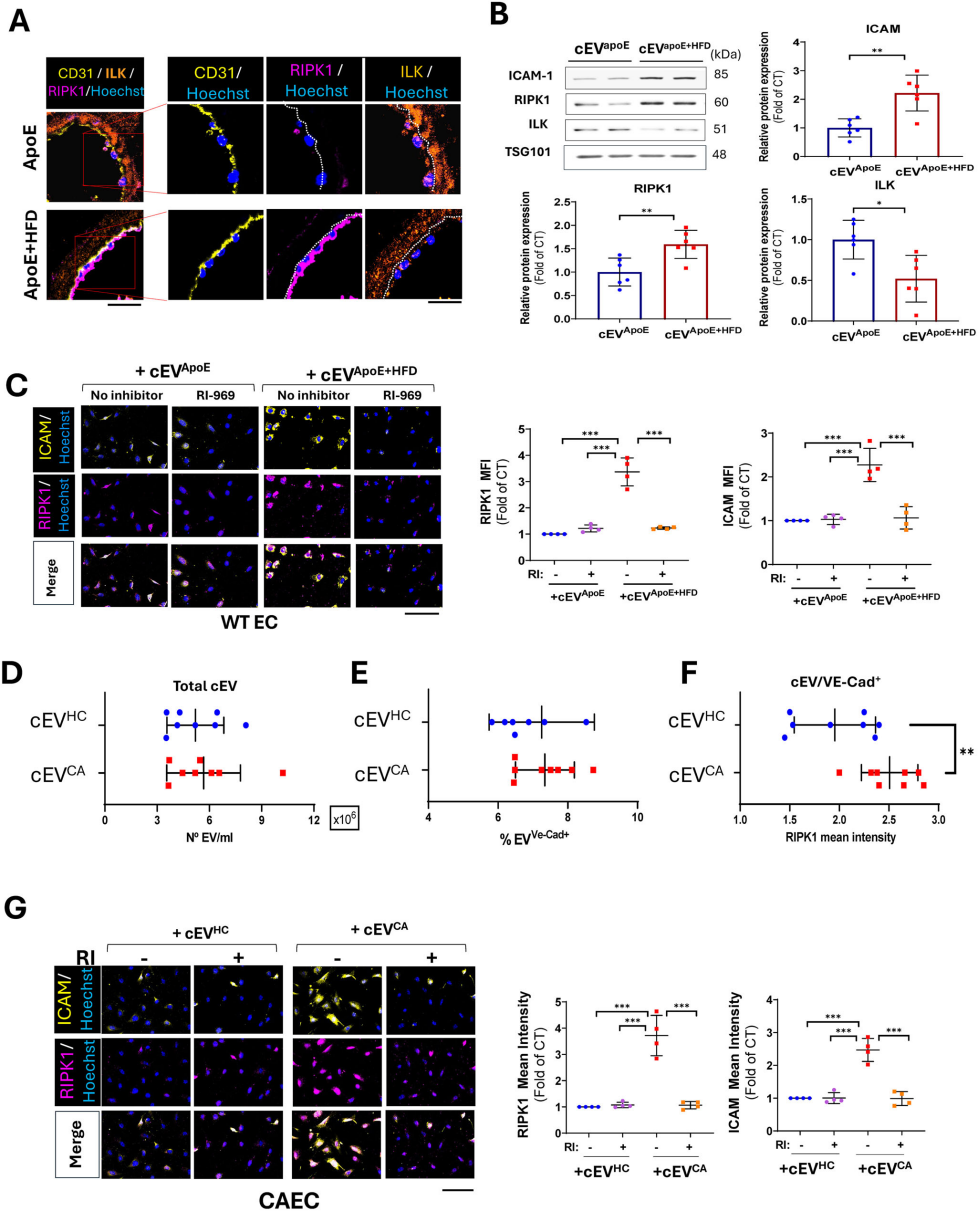
cEV from atherosclerotic mice and from patients with coronary atherosclerosis are enriched in RIPK1 and promote endothelial activation. (A) Representative immunofluorescence of aortic sections from ApoE−/− mice fed with regular (ApoE) or high‐fat diet (ApoE HFD) for 16 weeks, showing CD31 (yellow), ILK (orange) and RIPK1 (magenta) expression. Nuclei were counterstained with Hoechst (blue). Scale bar = 40µm and 20 Î¼m in the amplification. (B) Immunoblot analysis of ICAM‐1, RIPK1 and ILK in cEV from ApoE and ApoE HFD mice (cEVApoE and cEV ApoE HFD, respectively) (*n* = 6). (C) Wild‐type mouse aortic ECs were treated with cEVApoE and cEVApoE HFD in the presence or absence of RIPK1 inhibitor RI‐962(RI). Immunofluorescence analysis of ICAM‐1 (yellow) and RIPK1 (magenta) expression. Nuclei were counterstained with Hoechst (blue). Quantification of RIPK1 and ICAM mean intensities is shown on the right. Scale bar = 20µm (*n* = 4). (D) Concentration of cEV released per mL of plasma in healthy control individuals (cEVHC) and coronary atherosclerosis patients (cEVCA) (*n* = 8). (E) Flow cytometry quantification of VE‐ Cadherin+ cEV populations in plasma from healthy control individuals and CA patients. Results are expressed as a percentage of the Healthy Controls. (F) Flow cytometry analysis of RIPK1 enrichment in VE‐Cadh+ cEV from healthy and CA patients. (G) Human coronary artery cells (CAEC) were treated with cEV derived from healthy controls and CA patients in the presence or absence of RIPK1 inhibitor RI‐962(RI). Immunofluorescence analysis of ICAM‐1 (yellow) and RIPK1 (magenta) expression is shown. Nuclei were counterstained with Hoechst (blue). Scale bar = 20 µm. Quantification of RIPK1 and ICAM mean intensities is shown on the right (*n* = 4). Results are presented as mean ± SD. Statistical significance was determined using one‐way ANOVA or Student's *t*‐test, as appropriate. * *p* < 0.05, ** *p* < 0.01, *** *p* < 0.001.

